# Calreticulin: a multifunctional protein with potential therapeutic applications for chronic wounds

**DOI:** 10.3389/fmed.2023.1207538

**Published:** 2023-08-24

**Authors:** Andrew P. Sawaya, Nicole M. Vecin, Jamie L. Burgess, Nkemcho Ojeh, Gabrielle DiBartolomeo, Rivka C. Stone, Irena Pastar, Marjana Tomic-Canic

**Affiliations:** ^1^Wound Healing and Regenerative Medicine Research Program, Dr Phillip Frost Department of Dermatology and Cutaneous Surgery, University of Miami Miller School of Medicine, Miami, FL, United States; ^2^Faculty of Medical Sciences, The University of the West Indies, Bridgetown, Barbados

**Keywords:** calreticulin (CALR), chronic wounds, keratinocytes, wound healing, topical therapies

## Abstract

Calreticulin is recognized as a multifunctional protein that serves an essential role in diverse biological processes that include wound healing, modification and folding of proteins, regulation of the secretory pathway, cell motility, cellular metabolism, protein synthesis, regulation of gene expression, cell cycle regulation and apoptosis. Although the role of calreticulin as an endoplasmic reticulum-chaperone protein has been well described, several studies have demonstrated calreticulin to be a highly versatile protein with an essential role during wound healing. These features make it an ideal molecule for treating a complex, multifactorial diseases that require fine tuning, such as chronic wounds. Indeed, topical application of recombinant calreticulin to wounds in multiple models of wound healing has demonstrated remarkable pro-healing effects. Among them include enhanced keratinocyte and fibroblast migration and proliferation, induction of extracellular matrix proteins, recruitment of macrophages along with increased granulation tissue formation, all of which are important functions in promoting wound healing that are deregulated in chronic wounds. Given the high degree of diverse functions and pro-healing effects, application of exogenous calreticulin warrants further investigation as a potential novel therapeutic option for chronic wound patients. Here, we review and highlight the significant effects of topical application of calreticulin on enhancing wound healing and its potential as a novel therapeutic option to shift chronic wounds into healing, acute-like wounds.

## The pathophysiology and clinical burden of chronic wounds

Chronic non-healing wounds represent a major healthcare burden for patients and healthcare professionals (1–3). The main types of chronic wounds include venous leg ulcers (VLU), diabetic foot ulcers (DFU) and pressure ulcers (PU), and they all share a serious clinical burden due to the high incidence and recurrence, and associated complications (4). They are frequently associated with underlying conditions that include vascular disease, diabetes, and aging and the severity of chronic wounds as a serious medical disease is generally overlooked, despite a high mortality rate of 50% (5, 6). Five-year mortality rates for complications of DFUs such as minor and major amputations are 46.2% and 56.6%, respectively, compared to a pooled five-year cancer mortality rate of 31% (7). In addition, the excessive costs for diabetes care surpassed the costs associated with cancer care in 2017 (7). In addition, chronic wounds have a significant impact on quality of life due to lifestyle modifications, reduced motility, and social isolation and are associated with increased risk of depression, anxiety, and suicide (8, 9). Despite the severity of the clinical problem associated with chronic wounds, treatments remain limited and often not efficacious in restoring healing. Over 50% of DFUs and over 70% of VLU fail to heal depending on the wound size and duration (10–12), emphasizing the essential need to better understand the mechanisms that lead to chronic wounds for the development of new therapeutic options.

The physiological wound healing response progresses through a series of overlapping phases including hemostasis, inflammation, proliferation, and remodeling (13–16). Chronic wounds fail to progress through the phases of healing in a timely manner and are considered to be “stuck in a chronic inflammatory state,” contributing to inhibition of healing (17). This paradigm has been the longstanding rationale to therapeutically target the inflammatory process to curb inflammation. However, this has proven to be clinically unsuccessful. Recent studies have demonstrated an imbalance between pro-inflammatory and anti-inflammatory responses along with impaired function and signaling of inflammatory cells in DFUs and VLUs that fail to reach levels of acute healing wounds, rendering them incapable of progressing through the healing process (18–21). These previously unrecognized findings provide the basis for why therapies aimed at suppressing inflammation in chronic wounds has been clinically unsuccessful.

Keratinocytes play a crucial role in mediating the wound healing response and are among the first cells to respond to injury. Keratinocytes release several cytokines and factors that include IL-1β, TNFα, IL-6 and EGF that act in paracrine fashion to alert neighboring cells and local immune cells that the barrier has been breached (16, 22). In addition, these factors stimulate keratinocytes to acquire an activated phenotype (23). Upon activation, keratinocytes become hyperproliferative and migratory and begin to close the wound through re-epithelialization. The activated phenotype of keratinocytes is characterized by upregulation of the wound-inducible keratin genes, *Krt6, 16* and *17* along with upregulation of integrins and rearrangement of the actin cytoskeletal components to promote a migratory phenotype (22). Once keratinocytes have covered the wound, they begin the differentiation process to restore the epidermal barrier. In chronic wounds, keratinocyte activation is deregulated, in which they display a hyper-proliferative and non-migratory phenotype with hyperkeratosis and parakeratosis at the wound edge, further contributing to impaired barrier formation (16, 17, 24). The hyperproliferative, non-migratory epidermis is a hallmark of chronic wounds that is due to increased levels of the β-catenin/c-myc pathway (17, 25, 26). The overexpression of the β-catenin/c-myc pathway has profound effects on keratinocyte function that include downregulation of cytoskeletal components preventing a migratory phenotype along with (26) depletion of epidermal stem cells, contributing to inhibition of healing (25, 27).

Fibroblasts also play a crucial role in the wound healing process and are the main cell type responsible for the production of extracellular matrix (ECM) and granulation tissue as well as wound contraction, recruitment of immune cells, and angiogenesis (10, 28). Fibroblasts demonstrate phenotypic plasticity in response to wounding, which can contribute to proper healing or its pathogenesis (29–34). Fibroblasts regulate the wound healing response through secretion of signaling molecules and ECM proteins, adopt a transient contractile phenotype, and can serve as progenitors for specialized differentiated mesenchymal cells such as adipocytes (35, 36). Dysregulated fibroblasts in chronic wounds exhibit poor migration, proliferation, and ECM deposition, and increased senescence resulting in insufficient granulation tissue with increased fibrosis (17, 37–39). Aberrant Notch1 signaling has been shown to inhibit fibroblast growth and differentiation into myofibroblasts and diminished their potential for stimulating an angiogenic response (40). In addition, chronic wound fibroblasts exhibit dysregulated senescence, further contributing to impaired wound healing (41, 42). Furthermore, the hyperglycemic microenvironment of DFUs induces metabolic epigenetic memory that sustains DNA methylation patterns of genes critical to wound healing that contributes to impaired fibroblast migration as well as accelerates their senescence (37, 43, 44). In addition, chronic wound fibroblasts display dysfunctional lysosomal capacity and resultant impaired protein turnover, and aberrant TGF-β activity that causes impaired myofibroblast contraction and increased fibrosis (45, 46).

Immune cells play critical roles in all phases of the wound healing process (17). Neutrophils are among the first cell types to arrive at the site of injury that function to eliminate microbes to prevent infection and remove cellular debris (47, 48). Monocytes immediately follow and appear at the site of injury where they are activated in response to microbial products and inflammatory mediators that enable the transition to M1 macrophages, a pro-inflammatory phenotype that aids in preventing infection (49, 50). As the inflammatory response progresses, macrophages transition to an anti-inflammatory and pro-healing M2 phenotype that aids in repair of tissue (10, 50). In addition, the removal of apoptotic neutrophils by macrophages play an important role in augmenting the inflammatory response. They are also a rich source of pro-inflammatory cytokines (IL-1β and IL-6) and growth factors (FGF, EGF, and PDGF) that aid in initiating granulation tissue formation. We have previously demonstrated that immune cell recruitment of macrophages and neutrophils are deregulated in the wound edge of DFUs (19, 21). In addition, we identified transcriptional networks mediated by the FOXM1 transcription factor to be suppressed in DFUs that induce formation of neutrophil extracellular traps (NET), releasing cytotoxic proteins that can damage surrounding tissue, further contributing to inhibition of healing (19, 51). The inability of chronic wounds to clear the NETs along with decreased pro-healing M2 macrophages prevent chronic wounds from progressing through the wound healing process, causing them to be stalled in the inflammatory phase (52).

Effective treatments for chronic wounds remain limited due to the complexity of their pathophysiology, requiring a multifactorial approach that can target several aspects of the processes deregulated in chronic wounds. Here, we highlight the role of calreticulin (CALR) that functions as a multifunctional and versatile protein with remarkable pro-healing effects as a potential novel therapeutic option for chronic wounds.

## Calreticulin: overview

Calreticulin is a 46 kDa, high-capacity calcium-binding protein that is recognized for its role in regulating cellular processes in response to stress (53–56). The significance of CALR is underscored by the fact that knockout of calreticulin is embryonically lethal (57). It was initially identified as an endoplasmic reticulum (ER)-resident protein that resides within the lumen of the ER with two major functions: directing the proper folding of proteins, and homeostatic control of calcium levels in the cytosol and ER (54, 55, 58). However, later studies have demonstrated CALR to be localized in multiple compartments within the cell as well as in the extracellular matrix in which it has been demonstrated to be a potent pro-healing factor during wound healing (58–63). The initial discovery of CALR as a pro-healing factor came from studies that demonstrated purified hyaluronic acid (HA) accelerated wound healing in rodent models (64). However, it was later found that the pro-healing effects were mitigated by protease treatment, but not by hyaluronidase treatment, indicating an active protein to be in complex with HA responsible for the pro-healing effects (65, 66). Subsequent studies revealed, through purification and amino-acid sequencing, the active protein to be CALR.

The structure of calreticulin consists of three distinct functional domains that include the N-terminal domain, the middle P-domain, and C-terminal domain (55, 58). The N-terminal domain is the most conserved domain of CALR among different species and contains the signal sequence required for localization to the ER, whereas the middle P and C-terminal domains contain multiple high and low affinity calcium binding sites, respectively (58). The middle P-domain is rich in proline residues and consists of a pair of three repeated sequences that function in lectin-like chaperone activity termed A and B repeats with amino acid sequence PXXIXDPDAXKPEDWDE, and GXWXPPXIXNPXYX, respectively (55, 58). The C-terminal domain contains the canonical KDEL sequence necessary for the retrieval and retention of CALR in the ER. Together with its structural and functional homolog, calnexin, CALR directs the proper folding of proteins primarily through its lectin-like chaperone activity (67). Unlike CALR, calnexin is integrated within the ER membrane to coordinate proper folding of nascent proteins. Quality control of protein folding is carried out by recognition of oligosaccharides added to nascent polypeptides (68). The coordination between CALR and calnexin ensure proper folding of proteins by recognizing aberrant oligosaccharides and retaining the newly formed glycoprotein within the ER until the protein has acquired the proper conformation to enter the secretory pathway. Prolonged interaction with calreticulin/calnexin can activate the ER-associated degradation pathway (ERAD) to prevent accumulation of improperly folded proteins (69). Although studies on ER stress in chronic wounds are limited, several factors are known to induce ER stress that include hypoxia, oxidative stress and infection that are all associated with the pathophysiology of chronic wounds (70–72). ER stress has been shown to be elevated in diabetic and pressure ulcer mouse models, contributing to impaired healing (71, 72). Moreover, ER stress related genes were found to be upregulated in venous leg ulcers (70). However, it remains to be determined the role of CALR in mediating ER stress in chronic wounds.

CALR also has a critical role in regulating calcium homeostasis (73, 74). Calcium is a widely known important signaling molecule with multiple effects that include regulation of gene expression, cell adhesion and promoting keratinocyte differentiation (16, 75–77). Interestingly, CALR expression is greater in the suprabasal layers of the epidermis where calcium concentrations are higher relative to basal keratinocytes (60, 78), suggesting a role for CALR in regulating the differentiation process. Although the role of CALR in regulating epidermal differentiation remains unknown, it is interesting to reason that given its high expression level in the epidermis, particularly suprabasal keratinocytes, and its capacity to modulate calcium levels, it may play a previously unrecognized role in regulating epidermal differentiation. Calcium concentrations are three-fold higher in the lumen of the ER relative to the cytosol. The calcium storage capacity in the ER-lumen is enhanced by calcium-binding proteins that include CALR, Grp94, immunoglobulin-heavy-chain-binding protein (55, 58). Depletion of calcium stores from the ER-lumen can have profound effects that include impaired chaperone function and accumulation of misfolded proteins and blockage of the secretory pathway. In addition, calcium levels have a role in regulating expression of CALR in which depletion of intracellular calcium stores activates its gene expression (75, 79).

CALR is unique from most other ER-resident proteins in that its localization is not restricted to the ER (80). It is also found localized throughout the cell in intracellular compartments (e.g., nucleus, cytosol), cell surface as well as in extracellular compartments where it can interact and activate various signaling molecules to promote wound healing. Although cell surface CALR lacks a transmembrane domain, it complexes with its co-receptor low-density lipoprotein receptor-related protein 1 (LRP1) (81, 82). The LRP1/Calreticulin complex acts as a cell surface receptor for thrombospondin-1 (TSP-1) that is necessary to activate focal adhesion disassembly to promote migration (81–84).

Despite having the canonical KDEL ER-retention signal sequence, the exact mechanisms and how CALR localizes to multiple compartments remains poorly understood. Nevertheless, its localization to multiple compartments has demonstrated specific roles beyond functioning as an ER-chaperone protein during wound healing that include regulation of cell migration, focal adhesion disassembly, cellular proliferation, promoting phagocytosis, resistance to cell death and regulation of transcription factors (81–87), all of which are deregulated in chronic wounds. The extra-ER functions and application as a pro-healing factor in various wound healing models have been well established, making it an attractive therapeutic option for patients with chronic wounds (Table 1).

**Table 1:** Summary of the cellular components involved in acute and chronic wounds and the effects of calreticulin on each cell type in potentially reversing chronic wound pathophysiology.

Cell type	Timeline of wound healing	Acute wounds	Chronic wounds	Effect of calreticulin	Calreticulin receptor/protein interactions
Neutrophils	Inflammatory phase	Increased during inflammatory phase; proper balance between apoptosis and NETosis	Increased NETosis with low levels at wound edge and increased in wound bed	Enhances phagocytosis and clearance of apoptotic neutrophils	LRP1; PAI-1; azurocidin
Macrophages		Increased during inflammatory phase with proper balance of M1/M2 ratio	Increased M1 numbers with decreased M1–M2 transition	Promotes recruitment to wound; increases phagocytosis and cytokine production	LRP1; C1q and mannose binding lectin; LPS
T cells		Increased recruitment with high proportion of CD4^+^; increased epidermal T cell activation	Altered T cell populations with high levels of natural killer cell population and decreased epidermal T cell activation	Promotes recruitment, activation and priming of T cells; enhances cytolytic activity of T cells and natural killer cells	LRP1/TSP1/CD47
Endothelial cells	Proliferation/migration phase	Increased proliferation and migration to site of injury leading to increased angiogenesis	Decreased migration and proliferation leading to inadequate angiogenesis	Induces proliferation; promotes focal adhesion disassembly and cytoskeletal reorganization	LRP1/TSP1
Fibroblasts	Proliferation/migration phase	Increased proliferation and migration and ECM deposition; myofibroblast differentiation to promote wound contraction	Decreased proliferation and migration; increased senescence; increased fibrosis	Increases proliferation and migration into wound bed; promotes focal adhesion disassembly; increases TGFβ3, fibronectin and collagen production to produce granulation tissue; increases β1 integrin	LRP1/TSP1
	Remodeling phase				
Keratinocytes	Proliferation/migration phase	Proper balance between proliferation and migration; increased cytokine/growth factor production	Hyperproliferative and non-migratory; deregulated differentiation; aberrant growth factor signaling	Increases re-epithelialization and proliferation; induces fibronectin and α5 integrin	LRP1
	Remodeling phase				

## Calreticulin effects on keratinocyte function

Exogenous CALR treatment has shown significant effects in promoting keratinocyte migration in preclinical models of wound healing. *In vivo* studies using impaired healing models that include diabetic (db/db) mice models and steroid-treated porcine models, demonstrated enhanced rate of re-epithelization in CALR-treated wounds compared to controls (59, 60, 62). This was further supported by *in vitro* studies that showed CALR enhanced keratinocyte migration in a dose-dependent manner by a twofold higher migration rate than treatment with epidermal growth factor (60, 61), a potent stimulator of keratinocyte migration. Moreover, *in vivo* studies demonstrated CALR to be more effective in promoting wound healing than PDGF-BB, the only FDA approved recombinant protein for chronic wounds.

Although the exact mechanisms by which CALR promotes keratinocyte migration are poorly understood, it is possible that CALR may stimulate actin cytoskeletal signaling through activation of the LRP1 receptor (88). In chronic wounds, actin cytoskeletal signaling is deregulated due to increased caveolin-1 (Cav1) expression, contributing to inhibition of migration (89). Increased Cav1 expression results in increased RhoA activity, preventing directional migration (89). In addition, studies have demonstrated that deletion of CALR increased caveolin expression and endocytosis (90). Although the effects of CALR on Cav1 levels have not been investigated in the context of wound healing, signaling through the LRP1/Calreticulin complex has been shown to inhibit RhoA activity (88), providing a potential mechanism of CALR action in reversing Cav1 negative effects on keratinocyte migration.

CALR has also been shown to regulate steroid receptor activity and block their ability to regulate gene expression (87, 91, 92). Activation of steroid receptors by their cognizant ligands are known to play an important role in regulating the wound healing response, particularly glucocorticoids (GCs). GCs are well known potent inhibitors of wound healing that exert their effects through the GR (93–96). GC synthesis is increased in chronic wounds, contributing to inhibition of healing (89). CALR has been demonstrated to antagonize GR and prevent its ability to regulate gene expression as well as function as a nuclear export for GR (91, 92, 97), suggesting an additional pro-healing mechanism for CALR. Activation of GR was shown to exert profound diverse effects including inhibition of apoptosis, interferon-y signaling pathway, promoted terminal differentiation while inhibiting early differentiation markers, along with inhibition of keratinocyte migration (17, 24, 96). In addition, GR interacts with a variety of signaling pathways and transcription factors important in wound healing. GR has been shown to antagonize the epidermal growth factor (EGF) pathway (98), one of the most potent stimulators of keratinocyte migration. Inhibition of EGF signaling by GR resulted in suppression of the early wound-healing marker, keratin 6 (K6), resulting in impaired healing. Furthermore, GR activation leads to activation of the β-catenin/c-myc pathway resulting in a hyperproliferative epidermal phenotype, a hallmark of chronic wounds (26, 99). Moreover, activation of c-myc leads to epidermal stem cell depletion, further contributing to the inability of the tissue to properly respond to injury (27). In addition, GCs have been shown to inhibit deposition of ECM proteins such as collagen and cytoskeletal components preventing a migratory phenotype (93, 96), all of which CALR has been demonstrated to promote and may potentially reverse in a chronic wound setting. Although the role of CALR in inhibiting GR has not been demonstrated in the context of wound healing, the potential to reverse the wide-spread effects of GR activity in chronic wounds with CALR would provide a highly advantageous effect to shift chronic wounds to an acute-like healing wound.

Furthermore, chronic wounds exhibit deregulation of keratinocyte differentiation in which the early markers of differentiation, keratins 1 and 10, at the non-healing edge of chronic wounds were found to be suppressed, whereas the late differentiation markers involucrin and transglutaminase were upregulated, contributing to inhibition of healing (24). In normal wound healing, keratinocytes undergo calcium-dependent differentiation as they migrate upward, eventually terminally differentiating to make up the cornified, outermost layer of skin (17, 100). A major role of CALR is maintaining calcium homeostasis (101) and given that keratinocyte differentiation is calcium-dependent (77), it may play a critical role in potentially reversing the impaired differentiation process in chronic wounds. However, further studies are needed to elucidate possible effects of CALR on keratinocyte differentiation.

The potent effects of CALR in enhancing keratinocyte and fibroblasts migration in preclinical models of wound healing underscore its efficacy as a potential therapy for wound healing disorders. Further investigations into the mechanistic aspects that CALR has on keratinocyte function, that include targeting the wound healing inhibitors GR and Cav1, are needed to further demonstrate its potency as a potential therapeutic to reverse the effects of these factors in chronic wounds to stimulate healing.

## Calreticulin effects on fibroblasts

The effects of CALR studied in porcine and murine wound models demonstrated that CALR induced a tissue regenerative response that was evident by the presence of epidermal appendages and lack of scarring through regulation of fibroblast function (61, 63). In addition, CALR induced a dose-dependent increase in granulation tissue formation, producing granulation tissue with greater depth and cellularity and enhanced wound tensile strength (60, 62). Fibroblasts are responsible for granulation tissue formation for contraction of the wound bed and in chronic wounds this function is impaired (63, 102). Fibroblasts isolated from db/db mice and compared to fibroblasts isolated from nondiabetic wild-type mice demonstrated that CALR significantly enhanced migration and wound closure (59). This was further supported by *in vitro* wound healing assays with human fibroblasts cultured in hyperglycemic conditions that demonstrated significantly enhanced fibroblast migration after treatment with 50 ng/mL CALR (59). Moreover, CALR stimulated increased granulation tissue formation, induced fibroblast production of collagen I, fibronectin, α5β1 integrins and elastin through signaling mediated by TGF-β3 (63), all of which promotes fibroblast migration into the wound bed (61, 63). While TGF-β1 is known for its role in scar formation and fibrosis as well as its wound healing effects, TGF-β3 is antifibrotic and promotes reduced scarring, supporting anti-scaring effects of CALR (103, 104). Moreover, topical application of CALR showed increased proliferation of dermal fibroblasts with increased dermal cellularity and organized collagen fibrils (62). Interestingly, knockout of either CALR or LRP1 in fibroblast cell lines resulted in inhibition of migration in the presence of TSP-1 (85, 86), confirming the importance of this receptor complex in promoting migration.

## Calreticulin effects on stem cell function

Epidermal stem cells of the skin are located in at least three distinct lineages, namely, the hair follicle bulge region, the epidermal basal layer and the base of sebaceous glands (10). These cells play an important role in epidermal homeostasis, regeneration and contribute to wound repair (105). Other adult stem cells such as hair follicle dermal papilla and dermal sheath cells, melanocyte progenitors, bone marrow-derived stem cells, mesenchymal stem cells and adipose progenitors have also been reported to contribute to wound healing (106). Adult stem cells promote healing by speeding-up re-epithelialization, exhibiting plasticity, accelerating angiogenesis, and releasing paracrine signaling molecules (106). Depletion of stem cells caused by overexpression of β-catenin/c-myc pathway has been shown to contribute to delayed and impaired wound healing (26). Although the effects of CALR on adult stem cells in the context of wound healing is poorly understood, its role in developmental processes and modulation of embryonic stem cell fate decisions has been reported (107, 108). Studies have shown that CALR knockout leads to embryonic lethality in mice whereas this phenotype is rescued when CALR is constitutively expressed (109) thus proposing CALR as a key regulator of cardiomyogenesis and vital for controlled cardiomyocyte development (109). The effects of CALR on cardiac development have been shown to be mediated through indirect transcriptional activity regulation of nuclear factor of activated T cell (NFAT3) (57) and myocyte-enhancer factor (MEF) 2C (110) by affecting calcineurin activity. Impaired nuclear translocation of these transcription factors in CALR-null cells were reestablished upon re-expression of CALR (57, 110). Furthermore, CALR plays an important role in central nervous system development (111), osteogenesis (107) and chondrogenesis (108) and hematopoiesis (112, 113). Pilquil et al. demonstrated that CALR regulated a switch between osteoblast and chondrocyte lineages derived from murine embryonic stem cells via calcineurin/NFATC1 axis and GSK3β-deactivation (108). CALR has been shown to affect β-catenin associated pathways and Notch and phosphoinositide 3 kinase signaling all of which are important in regulating stem cell fate decisions (114–117). It is interesting to note that several of these pathways overlap with stem cell regulation during wound healing that include NFATC1 (118) and GR/β-catenin/GSK3β/c-myc (26, 27, 99), suggesting a potential role for CALR in the regulation of progenitor cells during wound healing, however further studies are needed. The diverse effects of CALR in promoting development and wound healing as well as its ability to affect known stem cell associated pathways and antagonize activity of GR/β-catenin/c-myc pathway suggests CALR may participate in modulating stem cell regulation, preserve and/or protect local and systemic adult stem cell populations. Further research on the precise mechanisms underlying CALR’s role in stem cells in general and keratinocyte stem cells could provide valuable insights for potential therapeutic strategies in chronic wounds.

## Calreticulin effects on immune cell response

CALR is predicted to play a role in the attraction and migration of many immune cells including monocytes (61), T lymphocytes (119), natural killer cells (120), and dendritic cells (121). A major component of the wound healing inflammatory response involves tight regulation of the type and number of immune cell infiltration (10). While increased immune cells are a normal and necessary part of the wound healing process, the location, appropriate activation, and duration are critical for proper wound repair. Chronic wounds are characterized by an extended, suboptimal inflammatory response, with significantly decreased levels of neutrophils and macrophages compared to normal healing wounds (19, 20). The role of CALR in macrophage recruitment was demonstrated in porcine studies which found that CALR treated porcine wounds showed increased wound healing and, unlike wounds treated with buffer or PDGF-BB, showed up to three times the number of macrophages in the granulation tissue that resolved once past the inflammatory phase of wound healing (60, 61). Interestingly, approximately 50% of the macrophages were sequestered to the microvasculature of the dermis rather than the extracellular matrix (62), though the role of CALR in this localization and its subsequent effect in wound healing are still unclear.

CALR has been found to be a critical signal for immune cell activation and cytokine production. Cell surface expression of CALR on monocytes increases during their lifetime while in neutrophils, CALR expression is diminished over time (122). Flow cytometry and ELISA analysis found CALR was released from the cell surface when neutrophils are activated (123). Neutrophils secrete azurocidin, which has been shown to bind to CALR and induce cytokine production in monocytes (124). A partial recombinant CALR fragment from mice was found to stimulate human monocytes and B cell cytokine secretion (125), as well as stimulate B cell differentiation into antibody secreting cells (126). Interestingly, stimulation of macrophages with recombinant CALR induced expression of TNFα and IL-6. These findings were further supported by utilizing an anti-CALR antibody that blocked their expression (124, 127). Moreover, treatment of mice with anti-CALR antibody reduced CXCL15, IL-6, IL-1β, TNF-α expression and recruitment of immune cells (127). In addition, CALR was reported to act as an opsonin for bacterial clearance by binding with LPS and other danger-associated molecular patterns (DAMPS) that triggered induction of cytokines production in macrophages, indicating CALR may function as a pathogen-associated molecular pattern (PAMPs) involved in the clearance of pathogens (128). As chronic wounds are characterized by deregulated immune cell response at the wound edge, it is of interest that CALR was found to increase immune cell activity, suggesting CALR may restore proper immune cell response in chronic wounds.

A major challenge with chronic wounds is deregulation of the immune cell response that traps chronic wounds in a prolonged and dampened inflammatory state. Neutrophils play a major role in wound healing and are deregulated in chronic wounds (19, 48). Chronic wounds are characterized by increased NET formation in which neutrophils decondense their chromatin and release their contents into the extracellular space that acts as nets to trap and maintain microbial infection from spreading (51, 129–131). However, excessive NETosis is detrimental to the wound healing process and diabetes has been shown to prime neutrophils to undergo NETosis and inhibit wound healing (129, 130). We found increased NET formation to result from suppression of the FOXM1 network in DFUs (51). Although CALR has shown some effects on regulating the immune cell response, its effects on neutrophils in the context of wound healing have not been fully explored. Given the pro-healing effects in preclinical models of diabetic wound healing, it would be interesting to establish the effects of CALR on regulating the neutrophil response and the potential to inhibit NET formation and restore neutrophil function in chronic wounds to promote a proper inflammatory response.

## Calreticulin effects on apoptosis and phagocytosis

Considering wound debridement is standard of care in the treatment of DFUs, the role of CALR in skin as a naturally occurring component of wound debridement through phagocytosis of apoptotic or infected cells is of considerable interest (61, 132). CALR has been shown to be a major regulator of immunogenic cell death (ICD) and acts as a key discriminator against other forms of cell death (133–135). ICD is triggered by specific inducers that lead to ER stress, the release and membrane exposure of CALR, recognition of DAMPs, and activation of immune cells that elicits antigen-specific inflammatory responses (133–135). Studies in the cancer field have shown the translocation of intracellular CALR to the cell surface is required for phagocytosis of apoptotic tumor cells by dendritic cells and induction of an effective immune response (136, 137), including priming of cytotoxic T cells (138, 139). CALR has been constitutively found on several cell surfaces which has been shown to induce phagocytosis when CALR is uncoupled from other phagocytic inhibitor molecules, such as CD47 (140). The loss of CD47 association led to not only apoptotic, but even viable erythrocytes and leukocytes phagocytosed by macrophages and nonprofessional phagocytes (132).

Removal of apoptotic cells is divided into three steps namely recruitment, engagement and engulfing (141). Phagocytes are first recruited to the apoptotic cells by “find-me” signals released by the cells. Engagement follows which occurs through binding directly or via bridging molecules in response to ‘eat-me’ signals on the apoptotic outer cell membrane. Phagocytes then engulf the apoptotic cells with the help of engulfment-receptors which engage directly with ‘eat-me’ signals (141). Cell-surface CALR has been shown to be vital for cellular response to ICD, apoptosis and efferocytosis (132, 133). It plays a key role as a bridging molecule in recognizing and removing apoptotic cells via the LRP1/C1q collectin/ficolin pathway by direct binding to the complement protein C1q, collectin, and ficolin proteins that recognize apoptotic cells (123, 142, 143). Binding of CALR to LRP1 has been shown to induce phagocytosis and pro-inflammatory responses (144). Moreover, both endogenous CALR (132) and exogeneous CALR secreted by phagocytes serve as ‘eat-me’ signals (145, 146) required for clearance by phagocytic cells along with others such as phosphatidylserine (147). CALR in conjunction with LRP1 has been shown to mediate efferocytosis (132) where CALR is present on the surface of the apoptotic cell to be taken up by the phagocyte expressing LRP1 in a trans configuration. This requires CD47/integrin-associated protein (IAP), a known “do not eat me” signal described for viable cells, to be down-regulated on the apoptotic cell to block engagement of CD47 with the cell surface signal regulatory protein-α (SIRP-α) Src homology 2 domain-containing protein tyrosine phosphatase substrate on the phagocytic cell.

Overexpression of CALR has been shown to result in heightened cellular susceptibility to apoptosis, whereas CALR-deficient cells from CALR knockout mice exhibited significant resistance to staurosporine-induced apoptosis. This resistance was accompanied by reduced cytochrome c release from mitochondria and diminished caspase 3 enzyme activity (74). In Drosophila studies, cell surface CALR is required for apoptotic cell phagocytosis by hemocyte-derived phagocytes and is blocked by CALR antibodies (148). Moreover, dead CALR-null mouse embryo fibroblast cells were not phagocytosed unless rescued by exogenous addition of CRT (132). These studies highlight the crucial role CALR plays in cellular responses to apoptosis and efferocytosis. Given that CALR is involved in these processes, it is likely that CALR may contribute to the wound debridement process of nonviable tissue, damaged, dead or unwanted cells and bacterial clearance by phagocytosis to resolve inflammation and promote chronic wound healing (61). These findings implicate CALR to have beneficial pro-healing effects through regulating apoptosis and removal of non-viable tissue/cells that is of considerable interest and needs further investigation.

## Conclusions and future directions

**Figure 1:**
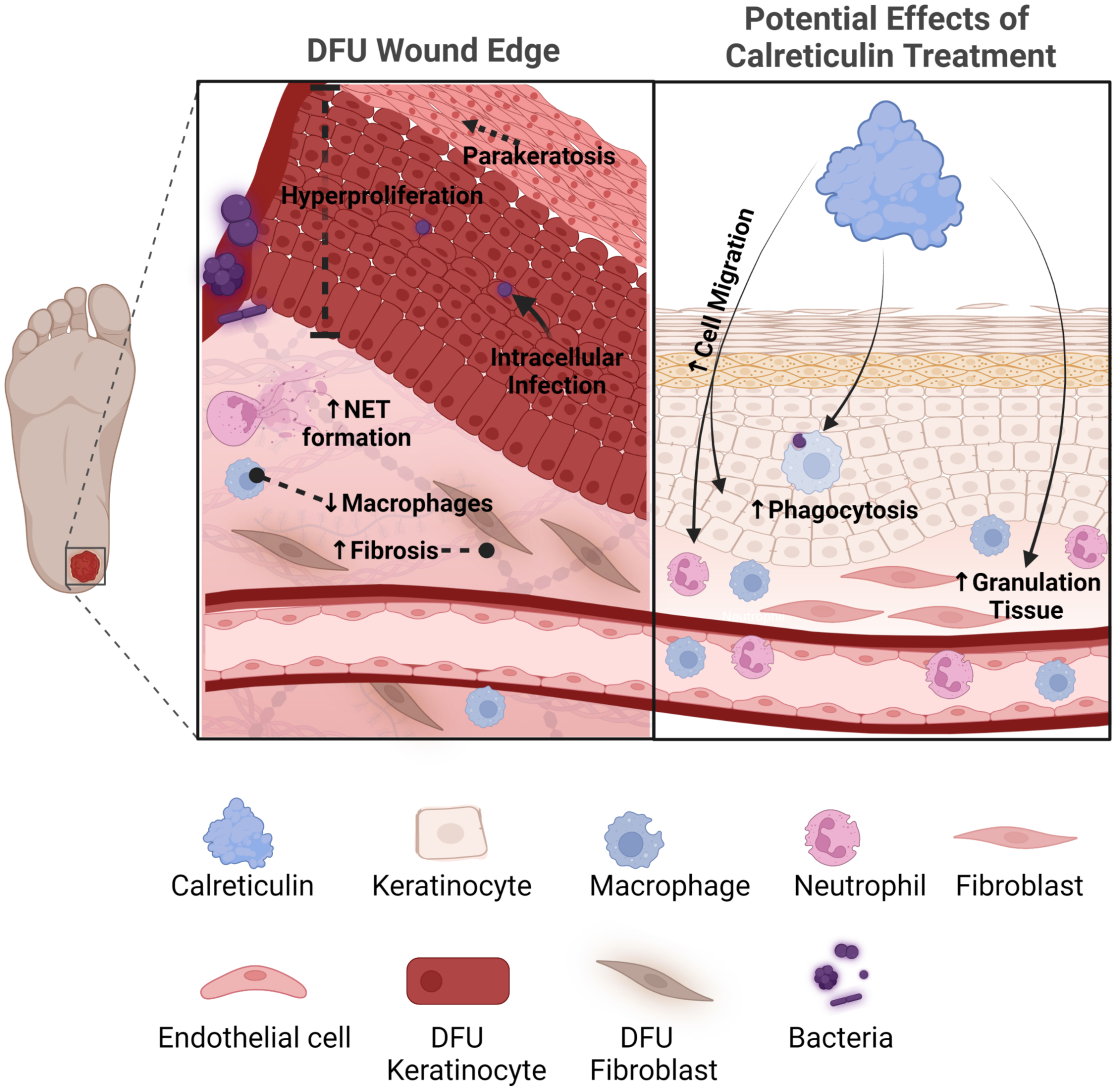
Calreticulin as a novel therapeutic with multiple pro-healing effects for the treatment of chronic wounds. Diabetic foot ulcers compared to acute healing wounds indicates the diverse effects calreticulin exerts on the wound healing process. Calreticulin enhances keratinocyte migration/proliferation, fibroblast function, granulation tissue and phagocytosis, all processes that are deregulated in DFUs that calreticulin may reverse to stimulate healing. Created with BioRender.com.

The various pro-healing functions of CALR makes it an attractive therapeutic option for patients with chronic wounds (Figure 1). Further studies are needed to determine the full effects of CALR action on additional processes of wound healing that include antimicrobial properties, angiogenesis, and effective topical delivery methods of CALR to patients.

The microbial burden of chronic wounds remains a major clinical challenge (149–151). Unresolved infection in chronic wounds further delays healing and contributes to the prolonged inflammation of the disease (10). Chronic wounds are characterized by a perturbed microbiome with increased bacterial biofilm formation (152). Although any direct antimicrobial effects of CALR during wound healing have not been determined, CALR has been shown to play a role in mediating the immune cell response during infection. Phagocytosis of pathogen-induced dying cells by dendritic cells is induced by exposure of CALR on the cell surface (153). There has been speculation that intracellular pathogens have found ways to avoid phagocytosis induced by CALR surface exposure (153), a speculation that is particularly relevant in DFUs considering recent studies have found that even DFUs with no clinical sign of infection harbor intracellular *Staphylococcus aureus* in keratinocytes (154). In neutrophils, CALR was found to bind an anti-bacterial peptide shown to have therapeutic activity against methicillin resistant *S. aureus* MRSA infected mice and stimulate superoxide anion production (124, 155). Conjugation of a recombinant CALR fragment with laminarin, a β-1,3-glucan, activated B cells and induced antibodies that prevented *Candida albicans* growth *in vitro* (156). However, further studies are needed to further characterize CALR as an antimicrobial in the context of wound healing.

Angiogenesis is an important aspect of wound healing that is deregulated in chronic wounds (157–159). Vasculature abnormalities are the result of impaired recruitment and function of endothelial cells and response to growth factors (e.g., VEGF), which contributes to decreased blood flow leading to ischemia and impaired healing (160). Studies on the effects of CALR on angiogenesis has shown mixed results, suggesting CALR may have context-dependent effects on the angiogenesis process. *In vitro* studies have demonstrated CALR increased proliferation of microvascular endothelial cells and VEGF production (60, 161). However, the effects of CALR on angiogenesis in tumor models have demonstrated that CALR undergoes N-terminal cleavage to generate the CALR fragment, vasostatin, that acts as an inhibitor of angiogenesis by preventing endothelial cell adhesion (162, 163). Nevertheless, it was demonstrated that the vasostatin fragment does not impair wound healing at concentrations used in tumor models (164). These findings indicate the context- and concentration-dependent regulation of CALR, further supporting the need to characterize the effects of CALR on angiogenesis in the context of wound healing.

CALR has also been shown to regulate several metabolic processes that include glucose transport, insulin receptor expression and iron metabolism (165–168), all of which play important roles in wound healing that are deregulated in chronic wounds. It was demonstrated that CALR regulates expression of insulin receptor and the GLUT1 transporter to maintain proper glucose levels (165, 167, 168). High levels of glucose is known to result in increased GLUT1 and insulin insensitivity (169). Under high glucose conditions, CALR promoted the degradation of GLUT1 mRNA resulting in overall downregulation of glucose transport into endothelial cells (165). In addition, CALR has been shown to act as a receptor for heparin in complex with LRP1 to prevent intracellular responses to high glucose concentrations (170). Moreover, the synergistic effects of CALR and heparin stimulated extracellular hyaluronan synthesis (170). In addition to regulating glucose metabolism, CALR has been shown to regulate iron metabolism (166), a process deregulated in chronic wounds in which excessive iron load contributes to impaired macrophage polarization and inhibition of healing (49). It was demonstrated that increased iron levels resulted in increased CALR expression in response to iron-induced oxidative damage in Caco-2 cells (166), a human intestinal epithelial cell line. Although the effects of CALR on metabolic processes in the context of wound healing remains to be investigated, it is interesting to speculate that CALR can have significant effects in restoring proper metabolic processes during wound healing that are deregulated in chronic wounds.

Another important aspect of future investigations into implementing topical CALR as a therapy for chronic wounds would be to evaluate the best course and formulation of topical delivery to patients. Exciting advances in nanotechnology-based drug delivery systems have been utilized as a delivery vehicle for topical based therapies. These include multiple matrix-based delivery systems such as electro-spun collagen or hydrogel-based delivery systems that may facilitate sustained topical delivery of CALR at the wound site and protects its activity from the high protease activity present in chronic wounds (171–175). Although topical application of recombinant proteins such as EGF and TGFβ, have shown success in several preclinical models of wound healing, the majority have failed to show efficacy in clinical trials, in part, due to the high degree of protease activity in chronic wounds (10, 176, 177). In addition, the receptors for these growth factors were found to be downregulated and mis-localized in the cytoplasm in patients with chronic wounds (46, 178), preventing patients from responding to exogenously applied growth factors. Although no clinical trials have been conducted with CALR in chronic wounds to date, these studies provide valuable lessons to learn from that can improve recombinant protein therapy in the chronic wound setting. Future work will be necessary to further characterize the receptors and factors that CALR interacts with in chronic wound tissue to assure full therapeutic potential of CALR. In addition, strategies to prevent degradation of CALR in the wound environment need to be investigated to protect its activity and allow CALR to exert its multiple pro-healing functions. These approaches will need to be tested in various *in vivo* models for determining an effective delivery system for CALR that can further support FDA applications for CALR as a topical treatment for patients with chronic wounds.

It is evident that the pathophysiology of chronic wounds is extremely complex that demands a multifactorial therapeutic approach that can target the multiple processes that are deregulated. Despite the advances made in understanding CALR effects in wound healing, many questions remain unanswered and the impact it has on wound healing may extend even further that may reveal additional novel mechanisms of CALR action in promoting wound healing. Given the multiple pro-healing effects of CALR that have already been established, topical CALR may provide a powerful therapeutic approach targeting multiple aspects of the chronic wound pathophysiology with maximum efficacy achieving the needed shift from a non-healing wound into an acute wound with healing properties.

## Author contributions

AS and MT-C: conceptualization, design, and review and editing. AS, NV, JB, GD, RS, NO, and IP: writing—original draft and writing—review and editing. All authors contributed to the article and approved the submitted version.

## Funding

This work was funded by the startup funds from the Dr. Phillip Frost Department of Dermatology, University of Miami Miller School of Medicine (to AS).

## Conflict of interest

The authors declare that the research was conducted in the absence of any commercial or financial relationships that could be construed as a potential conflict of interest.

## Publisher’s note

All claims expressed in this article are solely those of the authors and do not necessarily represent those of their affiliated organizations, or those of the publisher, the editors and the reviewers. Any product that may be evaluated in this article, or claim that may be made by its manufacturer, is not guaranteed or endorsed by the publisher.
